# The regulatory landscape of neurite development in *Caenorhabditis elegans*

**DOI:** 10.3389/fnmol.2022.974208

**Published:** 2022-08-25

**Authors:** Rasoul Godini, Hossein Fallahi, Roger Pocock

**Affiliations:** ^1^Development and Stem Cells Program, Department of Anatomy and Developmental Biology, Monash Biomedicine Discovery Institute, Monash University, Melbourne, VIC, Australia; ^2^Department of Biology, School of Sciences, Razi University, Kermanshah, Iran

**Keywords:** neurite development, transcription factors, neuronal guidance, gene regulatory landscape, *Caenorhabditis elegans*

## Abstract

Neuronal communication requires precise connectivity of neurite projections (axons and dendrites). Developing neurites express cell-surface receptors that interpret extracellular cues to enable correct guidance toward, and connection with, target cells. Spatiotemporal regulation of neurite guidance molecule expression by transcription factors (TFs) is critical for nervous system development and function. Here, we review how neurite development is regulated by TFs in the *Caenorhabditis elegans* nervous system. By collecting publicly available transcriptome and ChIP-sequencing data, we reveal gene expression dynamics during neurite development, providing insight into transcriptional mechanisms governing construction of the nervous system architecture.

## Introduction

Animals have developed nervous systems to enable the transmission of information over long distances. Information transfer between neurons relies on neurite projections called axons and dendrites. Correct neurite development is therefore critical for efficient signal transduction between neurons within a neuronal circuit. During development, neurites are guided to their targets by attractive and repulsive cues from surrounding cells/tissues and the extracellular matrix (ECM) (Adler et al., 2006; Wang et al., 2008; Dong et al., 2016; Miller and Suter, 2018). Many conserved ligands and receptors involved in neurite guidance have been identified over the last three decades (Tables 1, 2). However, diverse neuron sub-types within a nervous system have specific neurite projection patterns that are guided by distinct gene expression programs. Hence, neurite development requires precisely controlled spatiotemporal expression of intrinsic and extrinsic factors.

**TABLE 1:** Ligands that control *C. elegans* neurite development.

Signal type	Gene	Reference	Signal type	Gene	References
**Ephrin**	***vab-2***	Torpe and Pocock, 2014	**Wnt**	***egl-20***	Pan et al., 2006
	***efn-2***	Torpe and Pocock, 2014		***cwn-1***	Pan et al., 2006
	***efn-3***	Torpe and Pocock, 2014		***cwn-2***	Kennerdell et al., 2009
	***efn-4***	Dong et al., 2016		***lin-44***	Hilliard and Bargmann, 2006; Kirszenblat et al., 2011
**TGF**	***unc-129***	Colavita and Culotti, 1998; MacNeil et al., 2009	**Semaphorin**	***smp-1***	Ginzburg et al., 2002; Dalpe et al., 2004
	***let-756***	Bulow et al., 2004		***smp-2***	
	***tig-2***	Baltaci et al., 2022			
	***tig-3***			***mab-20***	Roy et al., 2000; Dong et al., 2016
**Slit**	***slt-1***	Hao et al., 2001; Fujisawa et al., 2007	**Netrin**	***unc-6***	Kulkarni et al., 2008; Hao et al., 2010; Smith et al., 2012; Levy-Strumpf and Culotti, 2014
**ECM**	***madd-4***	Seetharaman et al., 2011	**Leukocyte cell-derived chemotaxin**	***lect-2***	Díaz-Balzac et al., 2016

**TABLE 2:** Membrane-bound proteins that control *C. elegans* neurite development.

Protein type	Gene	References	Protein type	Gene	References
**Receptors**	***cam-1***	Forrester et al., 1999; Chien et al., 2017	**Receptors**	***lon-2***	Blanchette et al., 2015
	***cfz-2***	Zinovyeva and Forrester, 2005; Song et al., 2010; Wang and Ding, 2018		***mig-1***	Pan et al., 2006
	***daf-11***	Coburn et al., 1998		***mom-5***	Pan et al., 2006; Levy-Strumpf et al., 2015
	***daf-1***	Unsoeld et al., 2013		***pat-3***	Poinat et al., 2002
	***ddr-2***	Unsoeld et al., 2013		***plx-2***	Nakao et al., 2007; Dong et al., 2016
	***dma-1***	Liu and Shen, 2012		***sax-3***	Fujisawa et al., 2007
	***egl-15***	Bulow et al., 2004		***sma-6***	Baltaci et al., 2022
	***eva-1***	Fujisawa et al., 2007; Chan et al., 2014		***unc-5***	Hamelin et al., 1993; Norris et al., 2014
	***ina-1***	Baum and Garriga, 1997		***unc-40***	Gitai et al., 2003; Norris and Lundquist, 2011; Xu et al., 2015; Zhou et al., 2020
	***lad-2***	Wang et al., 2008			
	***lin-17***	Kirszenblat et al., 2011		***vab-1***	George et al., 1998; Zallen et al., 1999
	***lin-18***	Pan et al., 2006		***vem-1***	Runko and Kaprielian, 2004
**Cadherins**	***hmr-1***	Broadbent and Pettitt, 2002	**IgCAM (immunoglobulin family cell adhesion molecules)**	***rig-6***	Katidou et al., 2013
	***cdh-4***	Schmitz et al., 2008		***sax-7***	Wang et al., 2005; Ramirez-Suarez et al., 2019
	***fmi-1***	Steimel et al., 2010; Najarro et al., 2012; Unsoeld et al., 2013		***wrk-1***	Boulin et al., 2006
**Heparan sulfate proteoglycans**	***sdn-1***	Rhiner et al., 2005	**C-type lectin-like**	***clec-38***	Kulkarni et al., 2008

Transcription Factors (TFs) are key regulators of gene expression that control multiple processes of neuronal development, including polarization, migration and neurite guidance (Shirasaki and Pfaff, 2002; Santiago and Bashaw, 2014; Hayashi et al., 2015; Masserdotti et al., 2016). TFs regulate neurite development by controlling signaling, cell-adhesion and junction molecules, and cytoskeleton modifiers (Nobrega-Pereira and Marin, 2009; Santiago and Bashaw, 2014; Masserdotti et al., 2016). TFs can regulate neurite development cell-autonomously or regulate the expression of cues and receptors from surrounding tissues (Rauthan et al., 2007; Yoshimura et al., 2008). The complexity and dynamic nature of the nervous system makes *in vivo* analysis of regulatory mechanisms governing neurite development challenging. Animal models have been used to study neurite development, including mouse (Kuwajima et al., 2017), rat (Fontanet et al., 2013), zebrafish (Koh et al., 2020), fruit fly (Contreras et al., 2018), and *Caenorhabditis elegans* (Chisholm et al., 2016).

**FIGURE 1:**
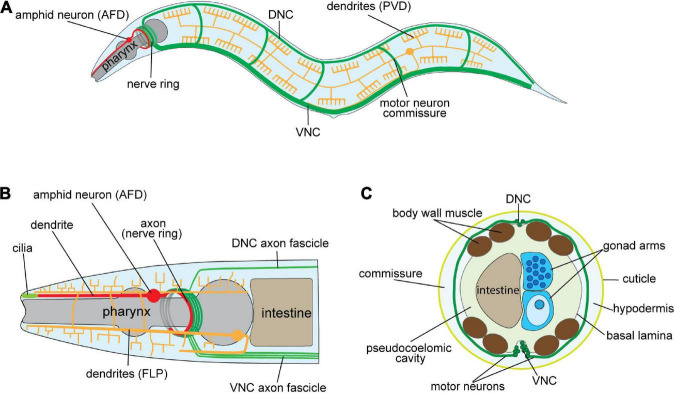
Architecture of the *Caenorhabditis elegans* nervous system. **(A)** Schematic of the adult *C. elegans* hermaphrodite nervous system (lateral view). Examples of major axo-dendritic processes: dorsal/ventral nerve cords, and circumferential commissures (DNC/VNC—green), amphid neurons, e.g., AFD (red), and the multi-dendritic PVD neurons (yellow) are shown. **(B)** Schematic of the *C. elegans* hermaphrodite anterior nervous system. Axons from the DNC, VNC and amphid neurons enter the nerve ring for integrating information and governing behavior. Amphid neurons (e.g., AFD) extend dendrites to the nose tip where cilia sense environmental cues. The FLP neurons have extensive dendritic arbors that, as with the PVD neurons in **(A)**, enable mechano- and thermo-sensation. **(C)** Schematic cross-section of the *C. elegans* mid-body showing longitudinal axon tracts, motor neurons circumferential commissures (DNC/VNC—green), and the location of other major tissues. The circumferential commissures are placed between the hypodermis and basal lamina.

The unparalleled detail of anatomical and molecular maps available for *C. elegans* renders it an exceptional model for studying fundamental requirements of nervous system development (White et al., 1986; Durbin, 1987; Cao et al., 2017; Cook et al., 2019; Packer et al., 2019; Taylor et al., 2021). *C. elegans* is a free-living nematode, with a short life span (∼3 weeks) and a transparent body that enables *in vivo* visualization of cellular structures at all stages of development. The *C. elegans* nervous system is small, containing 302 neurons and 56 glial cells (Figure 1; White et al., 1986). Importantly, neuronal positions are invariant and the synaptic connectivity (connectome) of these neurons has been mapped (Cook et al., 2019). Furthermore, single-cell transcriptomes of *C. elegans* neurons at distinct developmental stages are available (Cao et al., 2017; Packer et al., 2019; Taylor et al., 2021), enabling the study of how genes, including neurite development regulators and TFs, are expressed during neuronal development. Taken together, the depth of knowledge and ease of experimentation allow complex regulatory mechanisms required for neurite development to be studied in *C. elegans*.

Here, we reviewed all published neurite development-related TFs in *C. elegans*. In addition, we collected and integrated bulk and single-cell transcriptome data to extract expression patterns of ligands, receptors and TFs involved in neurite development (Cao et al., 2017; Packer et al., 2019; Papatheodorou et al., 2020; Sun and Hobert, 2021; Taylor et al., 2021). By analyzing ChIP-seq data we also constructed a network of potential TFs that bind upstream of genes encoding ligands and receptors that control neurite development (Gerstein et al., 2010). Thus, we provide an integrated view of neurite developmental regulators providing insight into mechanisms governing nervous system development.

## Neurites: The neuronal wiring system

Neurites are neuronal projections that transmit information within the nervous system and to non-neuronal cells and tissues (Cook et al., 2019). Neurites are classified as axons and dendrites that transfer information from and to the cell body (soma), respectively (Goaillard et al., 2019). In most vertebrates, axons are insulated by a protective myelin sheath that enables rapid electrical conductance, whereas some animals such as *C. elegans* lack such structures (Oikonomou and Shaham, 2011). Depending on the neuron type, dendrites may be a single projection or highly arborized to receive information from multiple neurons (Agostinone and Di Polo, 2015). Neurites directly transmit information to target cells through synapses that release specific neurotransmitters, or through electrical junctions that allow ion flow (Pereda, 2014; Sudhof, 2018).

**FIGURE 2:**
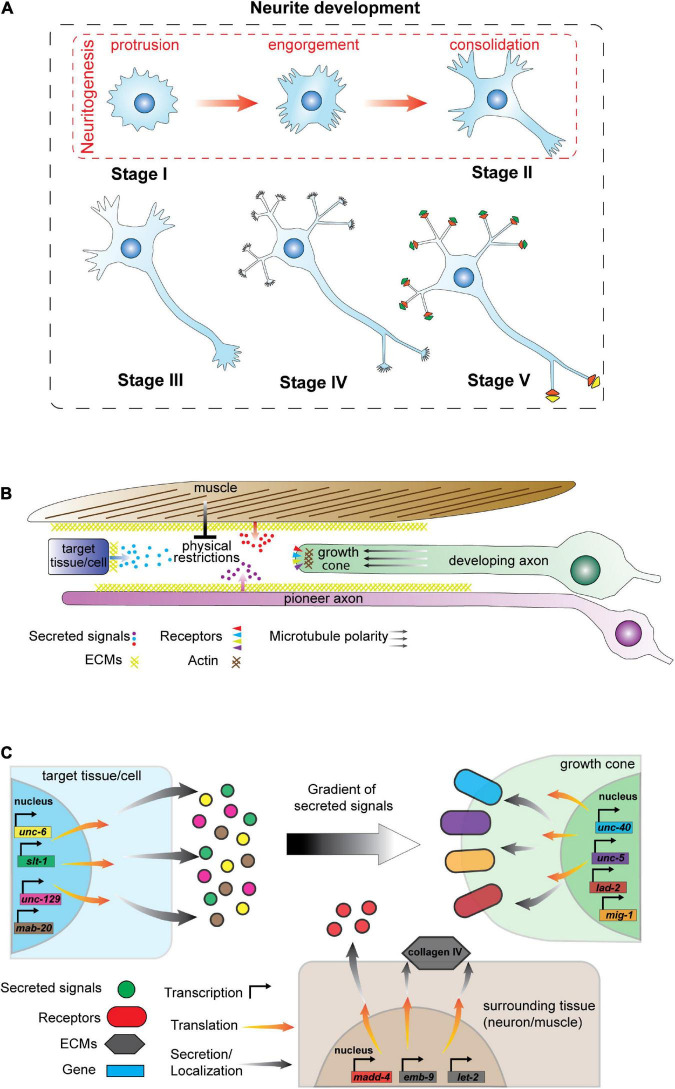
Neurite development and regulatory mechanisms. **(A)** Stages of neurite development *in vitro* in the absence of environmental cues. Neurite development consists of five stages: (I) lamellipodia or filopodia formation, (II) emergence of short and dynamic neurites, (III) axon establishment (IV) dendrite formation, and (V) formation of dendritic spines, axonal synapses and neuron circuits. Stage I and II are defined as neuritogenesis that has three phases: (1) protrusions that form lamellipodia or filopodia on the membrane; (2) engorgement of cytoskeletal components that enter lamellipodia and filopodia, and (3) consolidation of neurite growth cones by establishing a cylindrical cytoskeletal shaft. **(B)** Schematic of signaling events impacting *C. elegans* neurite development. Axo-dendritic guidance is controlled by signals released from other cells/tissues, the extracellular matrix (ECM) and physical barriers such as muscle. In addition, pioneer axons guide follower axons along specific tracks. **(C)** Schematic of signals and receptors that regulate axon development in *C. elegans*. Signal molecules (left) are synthesized by target and surrounding tissues/cells and are released from the cells or localized into the plasma membrane as a component of the ECM. Gradients of secreted signals guide growing axons. Elongating neurites (right) express receptors that localize at the growth cone to detect and respond to external signals. Neurons express distinct gene batteries and here we present candidate signals and receptors that may not expressed in the same cell at a given time.

Correct neurite development requires precise regulation of intrinsic cytoskeleton-driven mechanisms and extrinsic molecular interactions between cell-surface receptors and the surrounding environment. In the absence of external factors, neurite development of mammalian hippocampal cells in culture is classified into five main stages: (I) membrane ruffling by protrusion of cell-surface lamellipodia or filopodia, (II) emergence of short immature neurites, (III) axon establishment through stochastic growth of one neurite, (IV) conversion of all other neurites into dendrites, (VI) generation of dendritic spines and axonal synapses to establish a neuronal circuit (Dotti et al., 1988; Goslin and Banker, 1989; Figure 2A). The same scenario is expected to occur *in vivo*; however, initiation and growth of axons and dendrites is directed by gradients of guidance cues in the environment and by physical barriers (Hutter, 2003; Adler et al., 2006; Figure 2B).

Neurite development is therefore composed of projection, polarization and extension of neurites that are controlled by dedicated signaling pathways and cytoskeletal rearrangement. Detailed reviews on neurite developmental processes have recently been published: neurite polarity (Yogev and Shen, 2017; Armakola and Ruvkun, 2019); the cytoskeleton in neurite formation (Sainath and Gallo, 2015; Miller and Suter, 2018) and neurite development and repair (Richardson and Shen, 2019). Mechanisms governing axon guidance in *C. elegans* have also been reviewed by Chisholm and colleagues (Chisholm et al., 2016). In general, polarization determines how the cytoskeleton rearranges to direct projection and neurite formation (Schelski and Bradke, 2017), whereas, cytoskeletal stability regulates neurite growth by providing mechanical support, reviewed by Miller and Suter (2018). For example, neurite initiation, also known as neuritogenesis, begins with protrusion of lamellipodia or filopodia on the cell surface and movement of microtubules and other components into these structures, reviewed by Flynn (2013). Neurites are consolidated through cytoskeletal reorganization into a cylindrical structure at their base. Subsequently, polarization is shaped by the gradient(s) of cues that determine the direction of axo-dendritic growth (Schelski and Bradke, 2017; Miller and Suter, 2018).

Neuron polarization is the result of a combination of internal and external cues that impact cytoskeletal rearrangement (Miller and Suter, 2018). Internal cues are inherited from the existing apical-basal polarity of progenitor cells (Zolessi et al., 2006; Pollarolo et al., 2011), while extracellular cues can be produced by surrounding neuronal or non-neuronal tissues. External cues found to regulate neurite development in *C. elegans* are listed at Table 1. Polarity-related signaling molecules, such as UNC-34/Ena/VASP in *C. elegans*, and Cdc42 in mammals predominantly function by localizing at axonal growth cones and rearranging the cytoskeleton (Gitai et al., 2003; Schwamborn and Puschel, 2004; Fleming et al., 2010). UNC-34 is a downstream effector of the UNC-6/Netrin cue and its receptor UNC-40/DCC that are critical conserved regulators of axon guidance (Adler et al., 2006).

Following axo-dendritic growth, neurons communicate with each other by forming synaptic connections (Sudhof, 2018). Multiple processes are involved in synapse formation, including dendrite branching and pruning (Riccomagno and Kolodkin, 2015). Dendrite branching is a crucial step for establishing connections between different cells that also requires cytoskeletal rearrangement and cell surface molecule interactions, reviewed by Jin and Kim (2020). Additionally, neurite development requires other important processes including axon arborization (Gibson and Ma, 2011) and neurite pruning (Schuldiner and Yaron, 2015). With the latter, a critical step that removes superfluous neurites formed during development (Lu and Mizumoto, 2019).

In summary, neurite development is a multi-step process that can initiate during embryogenesis and continue over the lifespan of an organism. In *C. elegans* most neurons develop during embryogenesis (222 of the 302 neurons in the hermaphrodites), while others (80 of the 302 neurons) develop post-embryonically (White et al., 1986; Durbin, 1987). Here, we focus on axo-dendritic development in *C. elegans* and describe the mechanisms and TFs that regulate these processes.

## Genetic control of axo-dendritic development

Correct neurite development is controlled by coordinated intrinsic and extrinsic molecular mechanisms. In *C. elegans*, the majority of molecules identified to regulate neurite development were found by studying axon guidance. Multiple factors impact axon development including physical restrictions imposed by surrounding tissues, cues from other cells, cell surface molecules and receptors (Durbin, 1987; Baum and Garriga, 1997; Kim and Wadsworth, 2000; Levy-Strumpf and Culotti, 2014; Figure 2C). Tables 1, 2 lists the cues and membrane proteins shown to control *C. elegans* neurite development. In addition to these cues and receptors, there are multiple intracellular proteins important for cytoskeletal rearrangement and growth cone navigation that are not reviewed here (more details from Chisholm et al., 2016). Instead, we focus on the cues and receptors involved in the neurite development, with a focus on axon guidance.

Axon guidance cues can be secreted from surrounding tissues or presented within the ECM (Table 1). The ECM is a complex structure containing laminins, collagen IV and nidogen that provides a substratum for axon guidance by localizing guidance cues (Forrester and Garriga, 1997; Kim and Wadsworth, 2000; Huang et al., 2003; Kao et al., 2006). ECM components are sensed by receptors such as integrins (Baum and Garriga, 1997), dystroglycan (Johnson et al., 2006; Lindenmaier et al., 2019) and Discoidin domain receptors (Unsoeld et al., 2013) to control axon development. The ECM consists of multiple distinct proteins (Yue, 2014) and cells/tissues may express different combinations of ECM molecules. Additionally, post-translational regulation of ECM proteins impact axon guidance. For example, the prolyl 4-hydroxylase DPY-18 regulates HSN axon guidance by impacting the folding of collagen IV proteins encoded by *emb-9* and *let-2* (Torpe and Pocock, 2014). Therefore, the specific effects of ECM regulation on neurite development may be highly context- and structure-dependent.

Secreted guidance cues may act as axon guidance attractants or repellents. Most cues in *C. elegans* were identified in reverse and forward genetic screens for ventral nerve cord (VNC) and circumferential axon guidance defects (Kim and Wadsworth, 2000; Hao et al., 2001; Boulin et al., 2006). The *C. elegans* VNC axons are present in fascicles that extend in the posterior-anterior axis (White et al., 1986). Some VNC axons, known as pioneer axons, extend prior to others and provide a platform for follower axons in the same tract by producing extracellular cues, such as UNC-6/Netrin and SAX-3/Robo (Klose and Bentley, 1989; Wadsworth et al., 1996; Hutter, 2003). In contrast, ventral-dorsal circumferential axons are not guided by pioneer axons and grow in response to trophic cues that act as attractants or repellents. Trophic guidance cues are classified in different groups, including Netrins, Transforming growth factor-β, Wnts, Slits and Semaphorins. We will briefly discuss each group in the following sections.

UNC-6/Netrin and SLT-1/Slit are well-studied and conserved guidance molecules. UNC-6/Netrin is expressed by ventral cells and possibly forms a gradient to direct axon guidance (Kulkarni et al., 2008; Hao et al., 2010; Smith et al., 2012; Levy-Strumpf and Culotti, 2014). Depending on Netrin receptor expression, UNC-6/Netrin can be an attractive or repulsive cue. For example, UNC-40/DCC expression directs axon attraction toward the UNC-6/Netrin, whereas, expression of UNC-5, either alone or in combination with UNC-40/DCC, repels axons from high levels of UNC-6 (Norris and Lundquist, 2011). SLT-1/Slit, a cue expressed in dorsal muscle, is interpreted by receptors such as SAX-3/Robo and EVA-1 that coordinate dorso-ventral guidance of the AVM axon in parallel with UNC-6/Netrin (Hao et al., 2001; Fujisawa et al., 2007).

Growth factors, such as TGF-β ligands, are prominent guidance cues during axon development (Table 1). UNC-129/TGF-β is secreted by dorsal body wall muscle and directs axon guidance through unknown non-canonical TGF-β receptors (MacNeil et al., 2009; Baltaci et al., 2022). Notably, UNC-129 binds to the Netrin receptor UNC-5 and facilitates UNC-6/Netrin repulsive guidance by enhancing UNC-5 and UNC-40 signaling (MacNeil et al., 2009). The precise roles of growth factors in axon guidance have recently been reviewed by Onesto et al. (2021).

The *C. elegans* Wnt ligands EGL-20, CWN-1, CWN-2 and LIN-44 also regulate axon development (Hilliard and Bargmann, 2006; Pan et al., 2006; Kennerdell et al., 2009; Kirszenblat et al., 2011). EGL-20 and CWN-1 regulate anterior-posterior axon guidance of the AVM and PVM neurons by binding to Frizzled receptors MIG-1 and MOM-5 (Pan et al., 2006). While LIN-44 and the Frizzled receptor LIN-17 regulate axon and dendrite development of PLM and PQR neurons, respectively (Hilliard and Bargmann, 2006; Kirszenblat et al., 2011).

Some members of the Semaphorin family and ECM components are released from cells to guide axons. For example, MAB-20/Sema2 is a secreted semaphorin that interacts with PLX-2/Plexin and LAD-2/L1CAM to guide axons of the DA, DB and SDQL neurons (Roy et al., 2000; Wang et al., 2008; Dong et al., 2016). While ECM components, such as the metalloprotease MADD-4, are secreted from dorsal and VNC and cooperate with UNC-6/Netrin and SLT-1/Slit to control both muscle arm extension and AVM axon guidance following interaction with the UNC-40/DCC receptor (Seetharaman et al., 2011).

Neurite guidance proteins, expressed in neurons or surrounding tissues, must be precisely expressed to enable correct neurite growth. Such multi-layered regulatory processes involving multiple tissues require precisely controlled gene expression to support development of both pan-neuronal and neuron-specific neurite characteristics. These include fine navigation of neurites to establish synaptic connections, or the exit of neurites of specific neurons from nerve fascicles. The limited number of regulators compared to the diversity of the nervous system suggest that these regulators work in combinatorial patterns to regulate neuronal guidance decisions in a context-dependent manner. For example, SLT-1/Slit and UNC-6/Netrin cooperate in AVM ventral guidance such that removing both ligands causes more severe axon guidance defects compared to each single mutation (Hao et al., 2001). Notably, in *C. elegans* most studies of axon guidance have been performed on the VNC and circumferential axons and information is limited for more structurally complex regions such as the nerve ring. In the next section, we discuss how specific TFs regulate neurite development in *C. elegans*.

## Transcriptional regulation of neurite development

TF function in neurite development has been examined for both dendritic and axonal development. Here, we review the TFs with experimentally validated roles in neurite development in *C. elegans*. All TFs shown to regulate neurite development are listed in Table 3 but due to space limitations we only discuss the role of some key TFs below.

**TABLE 3:** TFs that control *C. elegans* neurite development.

Developmental process	Gene	Domain	Superfamily	Neuron Type	References
					
**Dendrite development**	***ahr-1***	Myc-type/PAS	HLH/PAS	S (PVD)	Smith et al., 2013
	***egl-44***	TEA/ATTS	TEA/ATTS	S (PVD)	O’Brien et al., 2017
	***egl-46***	ZF	ZF C2H2	S (PVD)	O’Brien et al., 2017
	***fkh-2***	FHD	Winged helix-like	S (AWB)	Mukhopadhyay et al., 2007
	***mec-3***	HD/ZF	Homeobox-like	S (PVD)	Tsalik et al., 2003; Smith et al., 2013
	***unc-86***	HD/POU	Homeobox-like	S (PVD, IL2)	Smith et al., 2010; Schroeder et al., 2013
**Axon projection/growth**	***ceh-10***	HD	Homeobox-like	I (ALA)	Altun-Gultekin et al., 2001; Van Buskirk and Sternberg, 2010
	***ceh-14***	HD/ZF	Homeobox-like	I/S (ALA)	Van Buskirk and Sternberg, 2010
	***ceh-17***	HD	Homeobox-like	I/M (DA, ALA, SIA)	Pujol et al., 2000
	***dmd-3***	DMD	DM DNA-binding	S (PHC)	Serrano-Saiz et al., 2017
	***egl-44***	TEA/ATTS	TEA/ATTS	M (HSN)	Desai et al., 1988
	***egl-46***	ZF	ZF C2H2	M (HSN)	Desai et al., 1988
	***egl-5***	HD	Homeobox-like	M (HSN, D-type)	Hartin et al., 2017
	***hlh-16***	Myc-type	HLH	I (AIY)	Bertrand et al., 2011
	***hlh-3***	Myc-type	HLH	M (HSN)	Doonan et al., 2008
	***lim-6***	HD/ZF	Homeobox-like	M (AVL, DVB)	Hobert et al., 1999
	***lin-11***	HD/ZF	Homeobox-like	I (AVG, D-type)	Hutter, 2003; Schmid et al., 2006
	***sem-4***	ZF	ZF C2H2	M (HSN, AVL)	Basson and Horvitz, 1996
	***unc-86***	HD/POU	Homeobox-like	M (HSN)	Olsson-Carter and Slack, 2011
	***zag-1***	HD/ZF	Homeobox-like/ZF C2H2	I/M (DA, DB, DD, VC)	Wacker et al., 2003
**Axon guidance**	***ahr-1***	Myc-type/PAS	HLH/PAS	S (AVM, SDQR)	Qin and Powell-Coffman, 2004; Smith et al., 2013
	***ast-1***	Ets	Winged helix-like	I (PVP, PVQ)	Schmid et al., 2006
	***ceh-14***	HD/ZF	Homeobox-like	I/S (ALA)	Kagoshima et al., 2013
	***ctbp-1***	ZF	NAD(P)/ZF	M (SMD)	Reid et al., 2015
	***fax-1***	NHR/ZF	ZF/NHR	I (AVK, HSN, PVQ)	Wightman et al., 1997; Much et al., 2000
	***ham-3***	SWIB/MDM2	SWIB/MDM2	M (HSN)	Weinberg et al., 2013
	***mab-9***	T-box/p53-like	T-box/p53-like	M (VNC)	Pocock et al., 2008b
	***mig-32***	ZF, RING-type	Zinc finger, RING-type	M, I (HSN, VD, PVQ)	Karakuzu et al., 2009
	***mls-2***	HD	Homeobox-like	S (ADF, AFD, AWC)	Yoshimura et al., 2008
	***mnm-2***	ZF	ZF C2H2	M (M2)	Rauthan et al., 2007
	***spat-3***	ZF	E3 ubiquitin-protein ligase RING1/RING2	HSN	Karakuzu et al., 2009
	***ttx-3***	HD/ZF	Homeobox-like	I (AIY)	Altun-Gultekin et al., 2001
	***unc-130***	FHD	Winged helix-like	M (DA, DB, DV, VD)	Nash et al., 2000
	***unc-3***	IPT	COE	M (HSN, DA, VA, VC)	Wightman et al., 1997; Prasad et al., 1998
	***unc-30***	HD	Homeobox-like	M (DD, VD)	Jin et al., 1994
	***unc-39***	HD	Homeobox-like	Unknown function (CAN)	Yanowitz et al., 2004
	***unc-42***	HD	Homeobox-like	S, M, I (ASH, HSN, PVQ)	Wightman et al., 1997; Brockie et al., 2001; Berghoff et al., 2021
	***unc-86***	HD/POU	Homeobox-like	M (HSN)	Olsson-Carter and Slack, 2011
	***vab-3***	HD/paired domain	Homeobox like/winged helix-like	S (ADF, AFD, AWC)	Yoshimura et al., 2008
	***vab-7***	HD	Homeobox-like	M (DB)	Esmaeili et al., 2002

Neurite development was grouped into dendrite development, axon outgrowth and axon guidance.

S, Sensory neuron; M, Motor neuron; I, Interneuron.

### Dendrite development

In *C. elegans*, most mechanistic information for dendrite development originates from studies of the PVD sensory neurons that develop post embryonically, and exhibit extensive and clearly visible dendritic branches (Figure 1A; Smith et al., 2010, 2013; O’Brien et al., 2017). PVD dendrites are classified into two groups: (i) pioneer dendrites that attach the epidermis, and (ii) commissural dendrites that fasciculate with motor neurons (White et al., 1986; Halevi et al., 2002; Tsalik et al., 2003). Several TFs regulate development of these dendritic structures. Among them, MEC-3, a LIM homeodomain TF, is a central regulator of PVD function and dendrite development (Tsalik et al., 2003; Smith et al., 2010). MEC-3 function is dose-dependent—with low levels driving dendritic branching of the PVDs, and high levels correlated with the simple dendritic morphology of the AVM mechanosensory neurons (Smith et al., 2013). AVM dendritic morphology is regulated by the aryl hydrocarbon receptor TF AHR-1, which elevates MEC-3 expression and suppresses MEC-3 target genes such as the HPO-30/Claudin protein (Smith et al., 2013). MEC-3 is also regulated by the POU TF UNC-86 and its loss phenocopies the *mec-3* mutant dendrite developmental defects (Xue et al., 1992; Smith et al., 2010). Additionally, UNC-86 is required for IL2Q dendrite arborization (Schroeder et al., 2013). In contrast, PVD commissural dendrite development is regulated by EGL-46, a zinc-finger TF, and its binding partner, EGL-44, a TEA/YAP domain TF (O’Brien et al., 2017). Interestingly, EGL-46 is itself a target of MEC-3 (O’Brien et al., 2017). Therefore, MEC-3 regulates dendrite development of the PVDs through two parallel pathways: HPO-30/Claudin for pioneer dendrites and EGL-46/EGL-44 for commissural dendrites.

Axons can also regulate dendrite development of other neurons. For example, the ALA axon regulates PVD dendrite development in a contact-dependent manner. Here, MIG-6/Papilin, an ECM protein, UNC-6/Netrin and UNC-40/DCC regulate ALA axon development in early developmental stages and this axon controls PVD dendrite development in the later stages (Ramirez-Suarez et al., 2019).

### Axon development

Most *C. elegans* studies of axon development were conducted on neurons with easily observable axons to facilitate mutant isolation (Figure 1, a general view of the *C. elegans* nervous system). These neurons include the ventral motor neurons (DA, DB, DD, and VD), VNC interneurons (AVG, PVQ, and PVP) and the HSN motor neurons (Jin et al., 1994; Pujol et al., 2000; Wacker et al., 2003; Doonan et al., 2008; Pocock et al., 2008b; Weinberg et al., 2013; Table 3).

TFs can regulate axon development both cell-autonomously and non-autonomously. TFs may also indirectly regulate axon guidance of some neurons by regulating pioneer axon development. For example, LIN-11 and UNC-30 affect VNC axon patterning by regulating AVG and PVP pioneer axon development, respectively (Hutter, 2003). TFs can also regulate development of a particular neuron when expressed in another neuron. For example, MNM-2, a zinc finger domain TF, controls axon guidance of the M2 neurons while its expression occurs in the M3, sister neuron of M2. Apparently, MNM-2 regulates axon guidance by functioning alongside genes involved in cytoskeleton or membrane dynamics, and Netrin/TGF-β signaling pathways (Rauthan et al., 2007). Glial cells also regulate axon development. Yoshimura and colleagues showed that CEPsh glial cells control AWC and AFD axon guidance within the nerve ring, dendrite development and nerve ring assembly (Yoshimura et al., 2008). Further, the MLS-2 and VAB-3 TFs, non-autonomously regulate axon guidance by regulating CEPsh development (Yoshimura et al., 2008).

TFs also regulate neurite developmental factors such as ligands, receptors and plasma membrane components in neuronal and non-neuronal cells. For example, UNC-130, a Forkhead TF, regulates motor neuron axon development through the dorso-ventral axis in parallel to the Netrin signaling pathway (Nash et al., 2000). UNC-130 represses expression of the UNC-129 TGF-β ligand in ventral, but not dorsal muscles, leading to a dorso-ventral biased gradient of UNC-129 and resulting in dorsal axon guidance (Nash et al., 2000). Further, UNC-42, a homeodomain TF expressed in 15 classes of neurons, regulates axon outgrowth and guidance of multiple neurons including ASH, AVH, AVA, AVD, and HSN (Wightman et al., 1997; Brockie et al., 2001; Berghoff et al., 2021). UNC-42 regulates axon guidance molecules such as UNC-6/Netrin in command interneurons, and LAD-2/L1CAM, RIG-6/IGCAM and NCAM-1 in a subset of UNC-42 expressing neurons (Berghoff et al., 2021). Further, the CTBP-1 transcriptional corepressor regulates multiple features of SMD axonal development, including outgrowth, guidance and termination (Reid et al., 2015; Sherry et al., 2020). CTBP-1 represses expression of SAX-7/L1CAM, a fibronectin type-III protein critical for neuronal development and maintenance (Wang et al., 2005; Ramirez-Suarez et al., 2019). It was revealed that ectopic overexpression of SAX-7 in neurons causes defects in the SMD axon development, revealing the importance of precisely controlling the expression of axon guidance molecules (Sherry et al., 2020).

TFs also regulate asymmetric and sex-specific axon development (Bertrand et al., 2011; Serrano-Saiz et al., 2017). In *C. elegans*, some neurons show asymmetric gene expression in either the left or right member of a bilaterally symmetric neuron pair (Johnston and Hobert, 2003; Bertrand et al., 2011; Cochella et al., 2014). Bertrand and colleagues found that the HLH-16 TF exhibits higher expression in some neurons on the left side compared to those on the right. The authors showed that HLH-16 controls axon projection of both left and right AIY interneurons, but the left AIY is more dependent on HLH-16 expression (Bertrand et al., 2011). This suggests a role for TFs in determining the asymmetric development of axon projections. Furthermore, specific TFs might also be responsible for sex-specific neurite development. Among neurons that are present in both hermaphrodites and males, some exhibit sex-specific connectivity and differential gene expression (Oren-Suissa et al., 2016; Serrano-Saiz et al., 2017). For example, sexual dimorphic development of neurites has been documented for the PHC sensory neurons where male axons are longer, and dendrites are retracted. This phenotype depends on sexual maturity, as both sexes show similar PHC anatomy in immature larval stages. DMD-3, a member of Doublesex TFs family, cell-autonomously regulates PHC neurite morphology (Serrano-Saiz et al., 2017). In turn, DMD-3 expression is itself regulated by the sex-determination pathway, through the TRA-1 TF that represses DMD-3 expression in hermaphrodites (Goodwin and Ellis, 2002; Serrano-Saiz et al., 2017).

In addition to TFs, other types of gene regulatory proteins, including chromatin remodeling and histone modifying enzymes, regulate neurite development. Polycomb Group proteins (PcG) are histone modifiers that control gene expression (Di Croce and Helin, 2013). In *C. elegans*, SOP-2, a polycomb protein, and SOR-3, a protein containing histone-binding domain MBT, show PcG-like function and repress the expression of homeotic genes (Zhang et al., 2003; Yang et al., 2007). Both SOP-2 and SOR-3 are required for the development of dopaminergic and serotonergic neurons and also control axon guidance of B-type ray neurons in the *C. elegans* male tail (Yang et al., 2007). MIG-32 and SPAT-3, members of RING-type domain-containing proteins, regulate HSN migration and axon development. In addition, MIG-32, a PRC1-like protein, controls axon guidance in the VD and PVQ neurons (Karakuzu et al., 2009). Another example is *ham-3* that encodes a subunit of the SWI/SNF chromatin-remodeling complex. HAM-3 controls serotonergic identity, migration and axon guidance of the HSN neurons (Weinberg et al., 2013). Therefore, epigenetic regulators and TFs control neurite development, likely by regulating the expression of specific genes.

The bias in study of neurite development toward those with clearly observable axons raises the question of how neurite development is regulated in the neurons within densely packed fascicles, such as those located in the *C. elegans* nerve ring. Another important question is whether pan-neuronal or neuron-specific gene regulatory systems control neurite development in all and specific neurons, respectively. As neurite development is controlled by multiple tissues, the existence of coordinated gene expression programs is likely. Therefore, TFs must orchestrate gene batteries in different cells to achieve this goal. To address these questions, analyzing the expression of the neurite development regulators (ligands, receptors and TFs) at a cell/tissue level throughout development would be a useful discovery tool for neurite specific developmental mechanisms. Here, we analyzed the expression of neurite development regulators through *C. elegans* development. Using bulk and single-neuron level transcriptomic datasets we examined how the expression of these genes change during neurite development.

## Expression patterns of neurite development regulators

**FIGURE 3:**
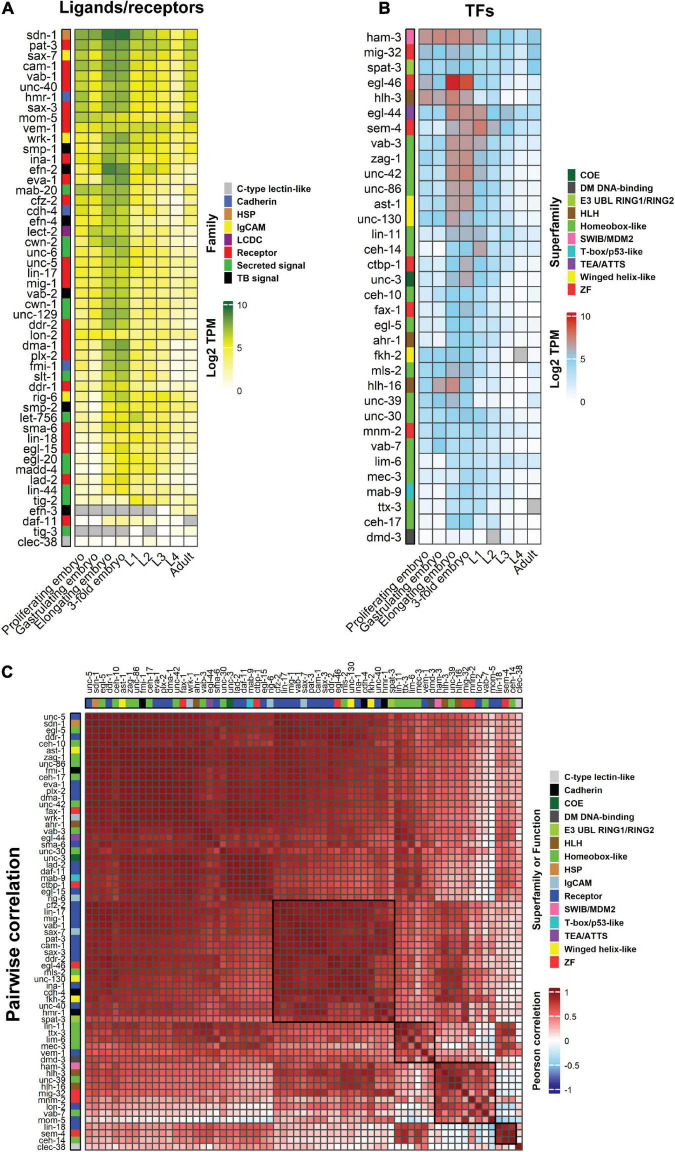
Heatmaps showing the temporal, quantitative and pairwise correlation of neurite development regulators. **(A,B)** Bulk expression of *C. elegans* ligands and receptors **(A)** and TFs **(B)** during embryogenesis, larval stages and adulthood. **(C)** Pairwise correlation between guidance receptors and TFs to identify expression correlations through animal development. Guidance ligands were excluded as they can be expressed in non-neuronal tissues. Genes are clustered according to Euclidean distance. Data are presented as Log2 TPM (transcripts per million) to normalize inter-sample differences. Legends show the ligand and receptor families or superfamily of TFs. Two histone modifier proteins, MIG-32 and SPAT-3, were also included in the TF lists. Gray cells = data not available. Black boxes delineate gene clusters. Gene expression values were obtained from Expression Atlas (ebi.ac.uk/gxa/home). HSP, heparan sulfate proteoglycans; LCDC, leukocyte cell-derived chemotaxin; TB, signal transmembrane-bound signal.

The availability of single-cell and whole animal transcriptome data throughout *C. elegans* development enables tracking of neuronal developmental regulator expression dynamics (Papatheodorou et al., 2020). Bulk expression of ligands (19), receptors (31 including other cell-surface molecules) and neurite development-related TFs (35) shows the majority of these genes are expressed in the elongating and 3-fold embryonic stages, which is consistent with the timing of neurite development (Durbin, 1987; Figure 3A). Generally, most neurite regulatory genes show higher expression during embryogenesis and lower levels at the final larval stage (L4) and adult stages (Figures 3A,B). However, only a few genes show a positive pairwise correlation with synchronous expression patterns during development (Figure 3C). For example, CEH-14, SEM-4 TFs and LIN-18 Wnt receptor show a high correlation of expression (Figure 3C). The other cluster of co-expressed genes includes the TFs HAM-3, HLH-3, HLH-16, MIG-32, MNM-2, UNC-39, and VAB-7, and the receptors LON-2 and MOM-5 (Figure 3C). To the best of our knowledge, there is no study showing a regulatory relationship between the receptors and TFs in these clusters. Therefore, it would be interesting to investigate possible biological connections between them. Bulk gene expression from whole animals does not however, represent neuron-specific expression patterns. To obtain a more precise view, we tracked the expression of neurite development regulators in single neurons.

**FIGURE 4:**
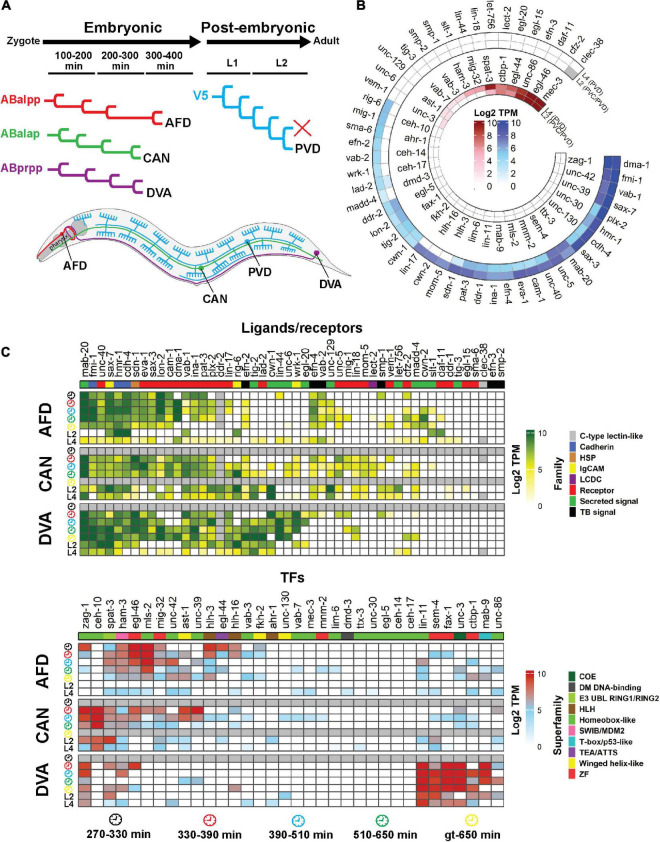
Single-cell expression analysis of neurite development regulators throughout development. **(A)** Four neurons (AFD, CAN, DVA and PVD) were selected based the availability of single-cell gene expression data and diversity of neuron type. The AFDs are sensory amphid neurons located in the head that extend anteriorly-directed dendrites and axons into the nerve ring (red). The CANs extend long neurites both anteriorly and posteriorly from the central cell body (green). DVA is a posterior sensory neuron that extends an anterior axon into the nerve ring (purple). PVDs are polymodal sensory neurons that extend elaborate dendritic arbors throughout the animal (blue). The AFDs, CANs and DVA develop embryonically while the PVD develops post-embryonically. **(B)** Expression of neurite developmental regulator ligands/receptors and TFs in the PVD (and PVC) neurons at the L2 and the PVDs at the L4 stage of development. The outer and inner heatmaps show the expression of ligands/receptors and TFs, respectively. **(C)** Single-cell expression of neurite development regulators in the AFD, CAN and DVA neurons during embryonic and larval development. Genes are clustered according to Euclidean distance. Data are present as Log2 TPM to normalize inter-sample differences. Legends show the family of the ligand and receptor proteins or superfamily of the TFs. Gray cells = data not available. Gene expression values were obtained from multiple studies: AFD, CAN and DVA at embryonic stages (Packer et al., 2019); AFD, CAN, DVA and PVD at L2 stage (Cao et al., 2017); AFD, CAN, DVA and PVD at L4 stage (Taylor et al., 2021). Clock faces denote time following bleaching of adult hermaphrodites. HSP, heparan sulfate proteoglycans; LCDC, leukocyte cell-derived chemotaxin; TB, signal Transmembrane-bound signal; gt, greater or equal to.

Single-cell *C. elegans* transcriptomes have been obtained from different stages of development, from embryonic through to larval stages (Cao et al., 2017; Packer et al., 2019; Sun and Hobert, 2021; Taylor et al., 2021). We focused on four neurons that extend neurites either in the embryonic (AFDs, DVA and CANs) or post-embryonic (PVDs) periods (Sundararajan et al., 2019; Figure 4A). More than half of the genes involved in neurite development are expressed at high levels during embryonic development of these neurons. The gene expression patterns of the AFDs, DVA and CANs are similar, though some genes exhibit a neuron-specific expression pattern. For example, the FAX-1 and CEH-10 TFs are highly expressed in DVA and CAN, respectively (Figure 4B). Interestingly, the MLS-2 and VAB-3 TFs, which control axon guidance of several neurons including the AFDs, are highly expressed in these neurons during embryonic development. However, these TFs regulate AFD axon guidance by functioning in cephalic sheath glia (Yoshimura et al., 2008). MAB-20/Sema2 semaphorin, which is a secreted cue, is expressed in all four neurons throughout development, suggesting that semaphorin may act as a common signal for neuron-neuron interactions. Likewise, the expression of many guidance receptor-encoding genes is detected in these neurons during embryogenesis, suggesting a role in neuron/neurite development. The PVD neurons, which extend dendrites post-embryonically (Sundararajan et al., 2019), express many of TFs including MEC-3, EGL-44, EGL-46, and UNC-86 that regulate PVD dendrite development (Smith et al., 2010, 2013; O’Brien et al., 2017; Figure 4C). Similarly, many guidance receptors are expressed in the PVD neurons at the L2 and L4 stages, highlighting their potential importance for dendrite development. For example, DMA-1 (a leucine-rich repeat protein), FMI-1/Flamingo, and SAX-3/Robo are required for PVD development (Liu and Shen, 2012; Hsu et al., 2020). SAX-7/L1CAM expression in hypodermal cells and the ALA neuron is also required for PVD development (Dong et al., 2013; Chen et al., 2019). However, this gene is also expressed in the PVDs, suggesting cell-autonomous and non-autonomous modes of action.

Taken together, many neurite development regulators are expressed during embryogenesis, when the majority of neurons develop. Interestingly, individual developing neurons show high expression of multiple neurite development regulators revealing the potential occurrence of combinatorial regulatory systems in each neuron. This hypothesis is supported by observations that removing individual neurite guidance regulators causes partially penetrant defects (Hao et al., 2001). Thus, our analysis of single-neuron transcriptome data through development reveals potential multifactor-controlled processes that requires regulatory tuning.

## Potential transcription factors involved in regulation of neurite development

**FIGURE 5:**
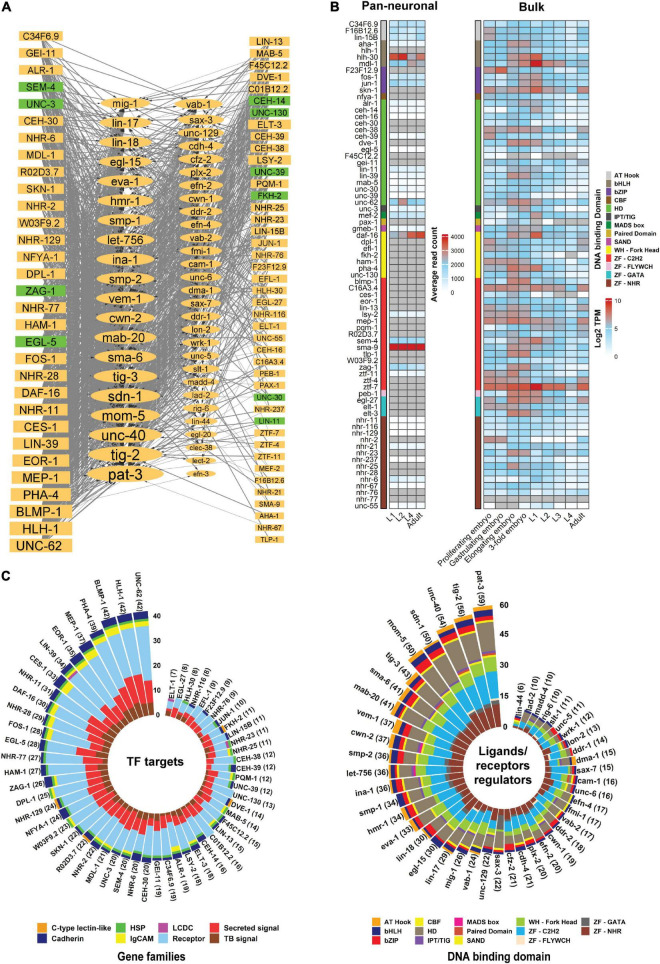
Transcriptional regulatory network of ligands and receptors that regulate neurite development.**(A)** Regulatory network of TFs with a binding peak upstream of genes that encode ligands and receptors involved in neurite development. The network was manually constructed by identifying statistically significant peaks (*q*-value < 0.01) within 2 and 5 kb upstream of the transcription start site (TSS) of each gene. For the genes with multiple isoforms the longest isoform was analyzed. Data was obtained from modENCODE (Gerstein et al., 2010; Yu et al., 2017; Li et al., 2020). The network was visualized *via* Cytoscape 3.7.1 (Shannon et al., 2003). Nodes represent genes in which the rectangles and ellipses show TFs and ligands/receptors, respectively. Node size relates to number of connections. Nodes in green are TFs with experimentally verified roles in neurite development. The edges show interactions between two nodes, where the thick and thin lines represent binding peaks at 2 and 5 kb from the TSS, respectively. Arrowheads depict TFs binding upstream of a gene. **(B)** Expression level of TFs (modENCODE) in the nervous system (pan-neuronal) or whole body (bulk) during animal development. Genes are not clustered. Gray cells = expression not detected. Pan-neuronal gene expression was obtained from Sun and Hobert (2021) and bulk gene expression was fetched from Expression Atlas (ebi.ac.uk/gxa/home). **(C)** Stacked bar plot showing target families for each TF and the superfamily of TFs binding upstream of neurite regulatory genes. TFs may have binding peaks upstream of multiple genes and a gene promoter can be occupied by multiple types of TFs. The left stacked bar plot shows the family of genes occupied by the TFs. The right bar plot represents which superfamily of TFs binding upstream of each ligand/receptor gene. In both plots, TFs and genes with more than 5 TF-gene interactions were visualized. The number next to the gene names shows the interaction counts. Interaction data were extracted from the network **(A)** and visualized using R language programming. HSP, heparan sulfate proteoglycans; LCDC, leukocyte cell-derived chemotaxin; TB, signal Transmembrane-bound signal.

Although genetic screens have identified several TFs (Table 3) that regulate neurite development, there are likely other potential regulators controlling this process. Analysis of ChIP-seq data enables the identification of putative TF target genes (Furey, 2012). Using this approach, we identified binding sites for TFs within the regulatory regions of neurite development regulators. The availability of ChIP-seq data (modENCODE) for multiple *C. elegans* TFs (74) enabled us to generate a regulatory network of TFs that bind upstream of genes encoding ligands and receptors involved in neurite development (Gerstein et al., 2010; Figure 5A). Among these TFs, EGL-5, ZAG-1, and UNC-3 are known to regulate neurite development (Wightman et al., 1997; Prasad et al., 1998; Wacker et al., 2003; Hartin et al., 2017). As one would predict, we found that multiple TFs can bind within 5kb upstream of genes encoding ligands and receptors (Figure 5A). Notably, the number of targets that are regulated by these TFs vary. Some TFs such as UNC-62, HLH-1, and BLMP-1 may bind to the upstream region of several ligand and receptor encoding genes, while others bind to specific regions, such as AHA-1, NHR-67 which have only one target gene each (Figures 5A,C). In addition, the upstream regions of genes encoding ligands and receptors are occupied with different TFs (Figure 5C). The expression of about half of these TFs is enriched in neurons throughout larval and adult stages (Figure 5B). However, expression level analysis shows no direct correlation between the expression of TFs and their targets. For example, despite the fact that HLH-1 and BLMP-1 bind to the upstream region of numerous target genes (Figure 5A), they do not show pan-neuronal expression (Figure 5B), however, low-level expression in specific neurons is possible. In contrast, DAF-16, a FOXO TF that binds to the upstream region of 30 ligand/receptor encoding genes, shows high neuronal expression during development (Figure 5B). This TF regulates multiple biological processes such as lifespan, metabolism, and axon regeneration (Lee et al., 2003; Murphy et al., 2003; Basu et al., 2021). Similarly, TFs with known functions in neurite development (Table 3), such as EGL-5, ZAG-1, UNC-3, and SEM-4, also have binding sites within the upstream regions of genes encoding ligands and receptors.

Promoters of ligand and receptor encoding genes may be occupied by multiple TFs. For example, *pat-3*, *tig-2* and *unc-40* promoters are occupied by over 50 TFs, however, the binding of these TFs could be cell-specific, or at particular developmental stages (Van Buskirk and Sternberg, 2010; Smith et al., 2013; Serrano-Saiz et al., 2017). In addition, some neurite development regulators function in non-neuronal tissues. For example, VAB-1/Eph receptor is required for epidermal morphogenesis (George et al., 1998). Therefore, coordinated cell/tissue transcriptional regulation of ligands/receptors by multiple TFs can orchestrate the function of these genes in distinct cellular contexts.

**FIGURE 6:**
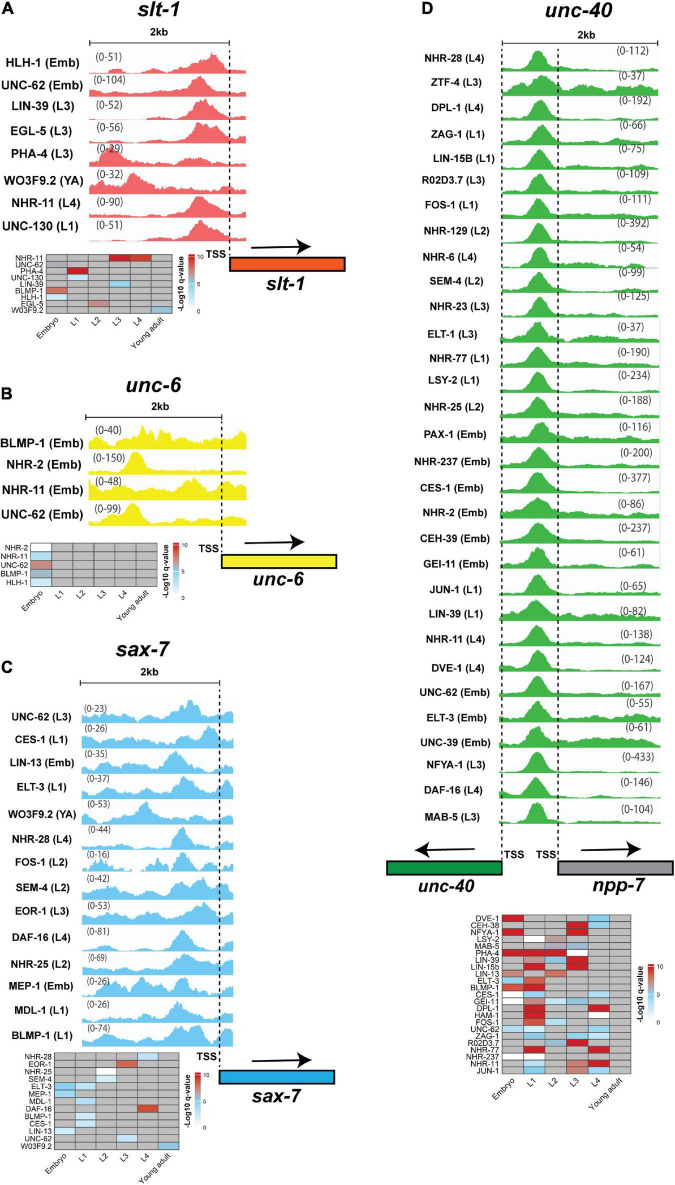
TFs binding peaks within 2 kb upstream of neurite development regulators. Genes encoding guidance cues **(A)** SLT-1, **(B)** UNC-6, and the guidance receptors **(C)** SAX-7, **(D)** UNC-40 were chosen to analyze ChIP-peak density. The heatmap below each group of peaks represent stages in which peaks were detected. Only a few TFs occupy 2 kb upstream region of *unc-6* and *slt-1*, however, the number of TFs that bind at 5 kb region is larger (Figure 5). Multiple TFs occupy the *sax-7* promoter. This gene has two short and long isoforms with the promoter region of short isoform occupied by 38 TFs (not shown). In contrast to the other genes, the *unc-40* promoter is occupied with several dozen TFs. Interestingly, the promoter region of *unc-40* overlaps *npp-7* promoter and as such some TFs may regulate the expression of both or either gene. All peaks are statistically significant (*q*-value < 0.01). The numbers on the peaks represent the scale of data within that region that shows the lowest and highest levels of the detected peaks within the region. Input levels are not shown or subtracted from the treatment peaks. The dashed lines show the location of TSSs. In the heatmaps, gray cells = no peak identified. Due to large number of peaks for *unc-40* and space limitation only some of the candidates are presented. The heatmap for *unc-40* includes genes with peaks at multiple stages. The peaks were visualized using Integrative Genomics Viewer 2.12.0 (Robinson et al., 2011). The data was obtained from modENCODE (Gerstein et al., 2010).

We found that some parts of the genome are hot spots for TF binding (Figure 6) (Araya et al., 2014; Joshi, 2014). However, linking these hot spots with gene expression and functionality need to be studied in more detail to identify how multiple TFs can cooperate with, or compensate for each other (Stefanakis et al., 2015; Leyva-Diaz and Hobert, 2022). In addition, promoter regions of some genes overlap. For example, the *unc-40* and *npp-7* promoters overlap with over 50 TFs binding within this region (Figure 6D). In this case, as both genes could be simultaneously expressed, it is challenging to identify which gene is the real target of each TF. Gene-specific regulation by each TF may require additional independent regulatory proteins, such as co-repressors and co-activators (O’Brien et al., 2018). Finally, some genes encode multiple isoforms that are controlled by alternative Transcription Start Sites (TSSs) (Rojas-Duran and Gilbert, 2012; Craig et al., 2013; Reyes and Huber, 2018). For example, *sax-7* encodes two short and long isoforms that regulate neuronal development and maintenance (Sasakura et al., 2005; Pocock et al., 2008a; Sherry et al., 2020). The upstream region of each isoform is occupied by different sets of TFs with several to dozens for longer and shorter isoforms, respectively (data not shown). This highlights the potential importance for transcriptional regulation of isoform-specific expression through binding of specific TFs.

Although the detection of TF binding upstream of a gene does not necessarily mean that the TF is a regulator of that gene, there is a possibility of regulation. To determine direct regulation, the expression of target genes in TF mutant backgrounds, TF-promoter binding assays and mutation of endogenous TF binding sites followed by ChIP-PCR would be necessary (Van Nostrand and Kim, 2013; Angelini and Costa, 2014; Pai and Gilad, 2014). Additionally, the difference in the number of regulators for different genes and its impact on the development and physiology must be investigated. Taken together, ligands and receptors involved in neurite development are potentially regulated by multiple factors.

## Evolutionary conserved transcription factors and potential connections to disease

Many TFs have DNA binding domains, such as zinc-finger and homeodomains, that are highly conserved across species (Narasimhan et al., 2015; Nitta et al., 2015). The majority of TFs involved in *C. elegans* neurite development have orthologs in other animal models such as fruit flies and mice. However, genes have different levels of conservation among species or may have multiple orthologs in other animals (Table 4). Interestingly, many *C. elegans* TF orthologs in mice are also required for nervous system development. For example, axon guidance is regulated by mouse Pax6 and Lhx2 TFs that are orthologs of worm neurite development regulators VAB-3 and TTX-3, respectively (Altun-Gultekin et al., 2001; Hevner et al., 2002; Lakhina et al., 2007; Yoshimura et al., 2008). This high level of evolutionary and functional conservation shows the importance of neurite development-related TFs. However, increasing complexity of the mammalian tissues may have caused alterations in specific TF functions from those observed in worms.

**TABLE 4:** Human orthologs of *C. elegans* TFs that control neurite development.

TF gene	Human ortholog (rank)		Disease-causing human gene	Disease description	Source
	High	Moderate			
***ahr-1***	AHR	AHRR			
***ast-1***	FLI1	FEV	FLI1	Amyotrophic lateral sclerosis	GWAS
***ceh-10***	VSX2	VSX1	VSX1, VSX2	Microphthalmia with coloboma	OMIM
***ceh-14***	LHX4	LHX3	–		
***ceh-17***	PHOX2A	PHOX2B	PHOX2A, PHOX2B	Fibrosis of extraocular muscles, neuroblastoma	OMIM
***ctbp-1***	CTBP2, CTBP1				
***dmd-3***		DMRTA1, DMRTA2, DMRT1, DMRTC1	DMRT1	Major depressive disorder	GWAS
***egl-44***	TEAD1, TEAD2, TEAD3		TEAD4	Narcolepsy with cataplexy	GWAS
***egl-46***		INSM1, INSM2			
***egl-5***		HOXC6, HOXB5, HOXA6			
***fax-1***	NR2E3		NR2E3	Enhanced S-cone syndrome	OMIM
***fkh-2***	FOXG1	FOXD3, FOXS1, FOXE3, FOXD2	FOXS1, FOXE3, FOXC1	Oppositional defiant disorder dimensions in attention-deficit hyperactivity disorder; Anterior segment dysgenesis	GWAS
***ham-3***		SMARCD1, SMARCD2, SMARCD3			
***hlh-16***		OLIG1, OLIG3			
***hlh-3***	ASCL1	ASCL2, ASCL4	ASCL1	Central hypoventilation syndrome	OMIM
***lim-6***	LMX1B	LMX1A			
***lin-11***		LHX1, LHX5			
***mab-9***	TBX10, TBX15				
***mec-3***		LHX1			
***mig-32***	PCGF3	PCGF5, PCGF1, PCGF6	PCGF6	Schizophrenia	GWAS
***mls-2***		HMX1, HMX2	HMX1	Oculoauricular syndrome	OMIM
***sem-4***	SALL1, SALL3	SALL2, SALL4	SALL3	Attention function in attention deficit hyperactive disorder	GWAS
***spat-3***	RING1, RNF2				
***ttx-3***	LHX2, LHX9				
***unc-130***	FOXD3, FOXD4	FOXD2, FOXD4L3			
***unc-3***	EBF1, EBF3	EBF2, EBF4	EBF3	Hypotonia, ataxia	OMIM
***unc-30***	PITX2	PITX3, PITX1	PITX2, PITX3, PITX1	Anterior segment dysgenesis, Bulimia nervosa	OMIM
***unc-39***		SIX4			
***unc-42***	PROP1		PROP1	Pituitary hormone deficiency	OMIM
***unc-86***	POU4F1, POU4F2, POU4F3	POU6F2, POU2F2			
***vab-3***	PAX6	PAX4	PAX6	Aniridia	OMIM
***vab-7***	EVX1, EVX2				
***zag-1***	ZEB1, ZEB2		ZEB1	Corneal dystrophy	OMIM

The orthologous genes and disease related to each gene was obtained from (fgr.hms.harvard.edu/diopt-dist) (Hu et al., 2011). High and moderate ranks are based on the score from integration of the results provided by individual ortholog tools. The high rank means the score is the best for comparing the gene of interest in the species A against B, and also reverse comparing of the species B against A. The moderate rank is obtained when the score is the highest in one of the comparisons of species. More details can be obtained from Hu et al. (2011, 2017).

Similarly, many of the neurite development TFs in *C. elegans* have human orthologs, with ∼50% of them associated with mental or neurodegenerative disorders (Table 4). Developmental defects in human neurons, particularly those related to eye disorders, such as Fuchs endothelial dystrophy, aniridia, and microphthalmia are linked to multiple TFs with a worm ortholog (Verma and Fitzpatrick, 2007; Nanda and Alone, 2019; Landsend et al., 2021; Table 4). Further, central hypoventilation syndrome and pituitary hormone deficiency are due to defects in the nervous system development caused by mutations in human orthologs of HLH-3 and UNC-42 (Correa et al., 2019; Trang et al., 2020; Table 4). In addition to developmental defects, neurodegenerative and psychological disorders also show association to orthologs of worm neurite development regulators. For example, amyotrophic lateral sclerosis and schizophrenia are associated to FLI1 (AST-1) and PCGF6 (MIG-32), respectively (Weinberger, 2019). However, the roles of these genes in human neuronal development are unknown. Psychological disorders, such as major depressive disorder and attention-deficit/hyperactivity disorder are caused by a combination of genetic and environmental factors (Otte et al., 2016; Posner et al., 2020; Table 4). These diseases may be caused by neurodegeneration or defects during development, including neurite guidance.

Conservation of TFs involved in neurite development across evolution suggest common regulatory mechanisms in the compact *C. elegans* and complex mammalian nervous systems. This similarity provides an opportunity to decipher fundamental mechanisms of neurite and nervous system development in model organisms, and to extend the knowledge to higher animals for potential therapeutic applications.

## Perspective

The nervous system contains complex neuronal circuits comprised of highly regulated neurite architecture. Correct nervous system development depends on precisely controlled gene expression patterns, and interactions of gene products in the surrounding environment. TFs are key regulators of gene expression that perform critical roles in neurite development. Most neurite regulators we examined show similarities in their expression patterns during embryonic and early larval stages in *C. elegans* (Figures 3A,B). Individual signaling pathways are shown to control neurite development (Hao et al., 2001, 2010; Fujisawa et al., 2007; MacNeil et al., 2009; Smith et al., 2012), however, multiple pathways also combine to provide robustness. For example, double mutants for *slt-1* (Slit) and *unc-6* (Netrin) exhibit increased defects in axon guidance compared to single mutation of these genes (Hao et al., 2001). We found that individual neurons express many neurite regulators at each developmental stage (Figure 4), suggesting the existence of multiple redundant mechanisms controlling neurite development. This hypothesis is highlighted in complicated axon growth behaviors. For example, axon guidance toward intermediate targets is controlled by precise expression of the Netrin signaling components UNC-5, UNC-6, and UNC-40 (Hedgecock et al., 1990; Wadsworth et al., 1996; Dickson and Zou, 2010). Another less explored example is the guidance of individual axons in a compact axon fascicle, such as nerve ring (Figure 1), where neuron-specific development of axons must be precisely controlled to enable synapse establishment. Hence, combinatorial signaling mechanisms seem to be inevitable, due to the existence of a limited number of the signal molecules and receptors that control neurite development.

Combinatorial interactions require highly regulated pan-neuronal and also neuron specific signaling mechanisms that are controlled by TFs. Neuron-specific regulatory mechanisms have been widely studied for neuron fate determination in *C. elegans* (Hobert, 2008; Stefanakis et al., 2015). Some TFs have been identified as “terminal selectors” that are essential for neuron fate determination throughout animal life (Hobert, 2008). Because of the neuron-specific features of neurites, such as growth to specific targets, there is a possibility that terminal regulator concepts for neurite development can be applied. Here, the limitation is that neurite development is a multi-tissue regulated process and, as such, requires co-regulation of genes in multiple tissues (Hao et al., 2001; Fujisawa et al., 2007; Norris and Lundquist, 2011). Further, the timing of TF and target expression is essential for proper neurite development. For example, the correct timing of UNC-86 expression is critical for axon initiation and activation of ventral guidance responses (Olsson-Carter and Slack, 2011). Alongside neurite growth, maintenance of neurite position is controlled by proteins such as SAX-7/L1CAM (Pocock et al., 2008a). The identification of regulatory mechanisms to ensure expression of “maintenance factors” may be critical for inducing axon growth following injury.

Combinatorial gene regulation requires the activity of multiple TFs (Reiter et al., 2017). We identified several TF binding sites present in the upstream region of genes encoding neurite guidance ligands and receptors, the relevance of which could be explored further using functional and mechanistic studies (Gerstein et al., 2010). Furthermore, the involvement of the reported TFs in neurite development has been studied in a limited number of neurons, with their regulatory roles in the majority of neurons unclear. In addition, TFs execute their functions *via* either protein-protein interaction with other factors or indirectly through other regulatory processes, suggesting a need to identify potential cooperative components for these TFs (Altun-Gultekin et al., 2001; Smith et al., 2013; O’Brien et al., 2017).

In addition to TF-dependent gene regulation, other gene expression regulatory mechanisms, such as epigenetic modification (Abay-Norgaard et al., 2020) and post-translational regulation *via* microRNAs (Zou et al., 2012; Hong et al., 2013; Pedersen et al., 2013) are involved in neuron development. Indeed, a combination of epigenetic modifications, transcriptional regulation by TFs and post-transcriptional modifications likely converge to precisely control nervous system development.

Finally, the dynamic and combinatorial behavior of neurite development regulators is reflected in the transcriptome, particularly from single-cell level analysis. This information may shed light on how to manipulate these regulators to overcome neuronal deficits and neurodegenerative decline.

## Author contributions

RG wrote the manuscript. HF and RP edited the manuscript. All authors contributed to the article and approved the submitted version.
